# Improving brain tumor segmentation with anatomical prior-informed pre-training

**DOI:** 10.3389/fmed.2023.1211800

**Published:** 2023-09-13

**Authors:** Kang Wang, Zeyang Li, Haoran Wang, Siyu Liu, Mingyuan Pan, Manning Wang, Shuo Wang, Zhijian Song

**Affiliations:** ^1^Digital Medical Research Center, School of Basic Medical Sciences, Fudan University, Shanghai, China; ^2^Shanghai Key Lab of Medical Image Computing and Computer Assisted Intervention, Fudan University, Shanghai, China; ^3^Department of Neurosurgery, Zhongshan Hospital, Fudan University, Shanghai, China; ^4^Radiation Oncology Center, Huashan Hospital, Fudan University, Shanghai, China

**Keywords:** masked autoencoder, anatomical priors, transformer, brain tumor segmentation, magnetic resonance image, self-supervised learning

## Abstract

**Introduction:**

Precise delineation of glioblastoma in multi-parameter magnetic resonance images is pivotal for neurosurgery and subsequent treatment monitoring. Transformer models have shown promise in brain tumor segmentation, but their efficacy heavily depends on a substantial amount of annotated data. To address the scarcity of annotated data and improve model robustness, self-supervised learning methods using masked autoencoders have been devised. Nevertheless, these methods have not incorporated the anatomical priors of brain structures.

**Methods:**

This study proposed an anatomical prior-informed masking strategy to enhance the pre-training of masked autoencoders, which combines data-driven reconstruction with anatomical knowledge. We investigate the likelihood of tumor presence in various brain structures, and this information is then utilized to guide the masking procedure.

**Results:**

Compared with random masking, our method enables the pre-training to concentrate on regions that are more pertinent to downstream segmentation. Experiments conducted on the BraTS21 dataset demonstrate that our proposed method surpasses the performance of state-of-the-art self-supervised learning techniques. It enhances brain tumor segmentation in terms of both accuracy and data efficiency.

**Discussion:**

Tailored mechanisms designed to extract valuable information from extensive data could enhance computational efficiency and performance, resulting in increased precision. It's still promising to integrate anatomical priors and vision approaches.

## 1. Introduction

Glioblastoma (GBM) is one of the most aggressive brain cancers among adults (1). Multi-parameter magnetic resonance imaging (MRI) provides valuable information for characterizing the size, invasiveness, and intrinsic heterogeneity of brain tumors (2, 3). Accurate delineation of GBM on multi-parameter MRI is crucial for clinical diagnosis and treatment, such as assisting surgical planning for maximum glioblastoma resection while preserving neurological function. However, the current clinical routine still relies on manual delineation, which is time-consuming and requires expert knowledge. There is a high demand for automatic brain tumor segmentation to enhance the efficiency of diagnostic procedures, facilitate surgical planning, and contribute to prognostic analyses (4).

In the last decade, there have been extensive studies on automatic brain tumor segmentation (5), and most of them are based on convolutional neural networks (CNNs) (6–8). However, due to limited receptive field, CNNs often struggle to capture long-range dependencies and global context (9, 10), potentially leading to inaccurate segmentation predictions. The recent success of transformer architecture in vision tasks (11, 12) has shown benefits in learning global contextual information. New network designs with vision transformers have emerged for medical image segmentation (13, 14) and achieved state-of-the-art (SOTA) performance in brain tumor segmentation (15–17). However, the supervised training of vision transformers typically requires a large amount of densely annotated images, otherwise there is a high risk of overfitting.

To combat the challenge of data scarcity in medical image segmentation, self-supervised learning (SSL) has proven to be a promising solution (18). In general, a pretext SSL task is designed to pre-train the network using unannotated data, and the learned encoder weights are further optimized in the downstream segmentation task. Since no manual annotation is needed for SSL, it can be applied to utilize large unannotated datasets. Recently, one of the most successful SSL frameworks is the masked language modeling (MLM), which has achieved great success in numerous natural language processing tasks with transformer-based architecture (19–21). Motivated by MLM, masked image modeling (MIM) was also proposed for pre-training vision transformers. In MIM, the model predicts masked image patches from unmasked patches. The prediction target can be either token features or raw pixel values of the masked patches. BEiT (22) utilizes a discrete variational autoencoder (dVAE) to transform all image patches into discrete tokens, which are then used to pre-train a vision transformer at the token level. However, tokenizing the image patches requires additional training of a dVAE. In contrast, He et al. (23) introduced the masked autoencoder (MAE), which randomly masks a subset of image patches and reconstructs the masked pixels from unmasked patches. The high masking ratio of MAE enables efficient pre-training of vision transformers with large annotated datasets. The success of MAE has motivated a series of variants in vision tasks (24–27) and applications in medical image analysis using MIM techniques. For instance, Tang et al. (28) utilized masked inpainting for the pre-training of a Swin UNETR (Shifted-window UNet transformer) in abdominal segmentation tasks. Chen et al. (29) compared multiple MIM approaches in abdominal segmentation. Zhou et al. (30) applied MAE pre-training with UNETR (UNet Transformer) and obtained performance gains in both abdominal and brain tumor segmentation.

**Figure 1:**
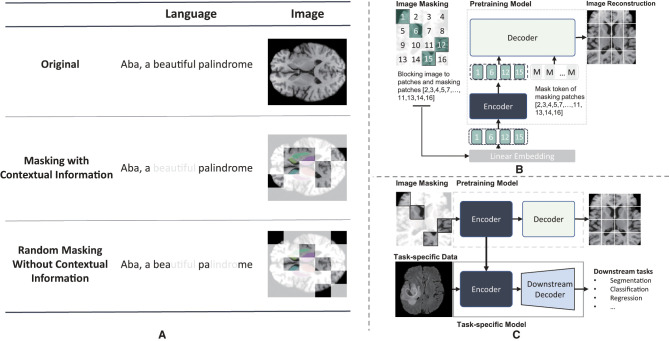
Illustration of maksed modeling pre-training and masked image modeling(MIM). **(A)** Different masking result of masked modeling pre-training. The light white regions represents the masked regions and the colored reigons of axial brain MR image represents different independent brain areas. **(B)** The pre-training illustraion of masked autoencoder. **(C)** the whole procedure of mim.

Building a masked image is a crucial step in MIM pre-training. As shown in Figure 1, the smallest masking unit of MLM, such as BERT (19), is typically the vocabulary, which preserves contextual information. However, MIM employs random masking, which can disrupt the spatial context and regions with the same semantic meaning, given the absence of the concept of words commonly observed in MLM. This, in turn, makes it challenging for the representation learning process to obtain high-quality pretrained network, especially when the masking ratio reaches a high percentage. Recently, several studies demonstrated that the masking strategy has a substantial effect on model performance in downstream tasks (31, 32). Although random masking is widely used, recent advances have shown that appropriate masking strategies can achieve better performance, such as region-based masking (33), attention-based masking (34), and adaptive masking (AdaMAE) (31). These masking strategies take the patch context into account, leading to more effective and efficient pre-training.

In the context of medical images, anatomical knowledge could help improve the pre-training. Huang et al. (35) incorporated the symmetry characteristics of brain structures into the pre-training by constructing symmetric positional encodings. However, few studies have integrated the more precise brain atlas (36) into the masking strategy. Inspired by the performance gains achieved by weighted masking strategies, we propose an anatomical prior-informed masking strategy for the MAE pre-training. We hypothesize that the tumor distribution among brain structures can guide the MAE pre-training, therefore improving the downstream brain tumor segmentation. To achieve this, we analyze the tumor occurrence in the SRI-24 space and establish an anatomical prior-informed probability map for image masking. This strategy allows us to select more informative patches for MAE pre-training. By combining the data-driven MAE with anatomical knowledge, we aim to improve the accuracy and data-efficiency of brain tumor segmentation.

In this study, our contributions are as follows:

(1) An anatomical prior-informed masking strategy is proposed to enhance the pre-training of masked autoencoder. This strategy is designed to preserve contextual information in 3D medical images and allows the pre-training process to concentrate on regions that are more relevant to the downstream segmentation task.
(2) By incorporating prior-informed weighted sampling, we construct an anatomical prior-informed masked autoencoder, referred to as API-MAE. This self-supervised pre-training approach utilizes 6,415 skull-stripped brain T1 MR images and combines data-driven reconstruction with anatomical priors.
(3) Inheriting the pretrained encoder weights, our method demonstrates superior performance in the downstream segmentation task on the BraTS21 dataset, outperforming several transformer models and surpassing state-of-the-art self-supervised learning methods. Subsequent experiments demonstrate that our method exhibits greater efficiency compared with a regular masked autoencoder and maintains a satisfactory trade-off between segmentation accuracy and computational consumption.

## 2. Methodology

### 2.1. Overview of proposed method

**Figure 2:**
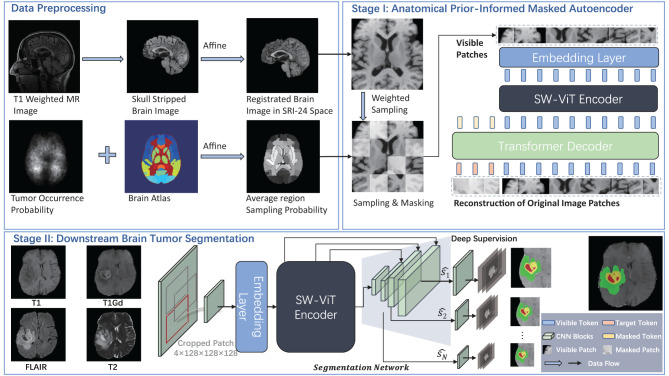
Overview of our proposed method.

We propose a novel masking strategy for improved MAE pre-training and downstream brain tumor segmentation in MRI. As shown in Figure 2, our proposed method consists of two stages: (1) pre-training a masked autoencoder with anatomical prior-informed masking strategy on the unannotated dataset and (2) transferring the pre-trained weights of the encoder and fine-tuning the segmentation network on the annotated dataset.

### 2.2. Statistical analysis of tumor occurrence

#### 2.2.1. Registration to standard brain template

To represent the anatomical priors, we first align all images with the standard brain template. The DICOM image data are transformed into Nifti format, and the brain is extracted using FSL tools (37). After that, we transform each image into the SRI-24 standard space (36) *via* affine registration. Using the optimized affine transformation matrix M^*^, all images are aligned in the SRI-24 space.


(1) $$\begin{array}{l} M^{*}=\underset{M}{\text{arg min}}\;C(I_{f},\mathrm{Affine}(I_{m};M))\\ \;I=\mathrm{Affine}(I_{m};M^{*}) \end{array}$$

where *I*_*m*_ represents the moving image, which corresponds to the MRI image of each sample. The fixed image, denoted as *I*_*f*_, refers to the T1 template of the SRI-24 standard space. In this study, the operation *C*(*I*_*m*_, *I*_*f*_) represents the cost function used to quantify disparities between the fixed image *I*_*m*_ and the moving image during the registration optimization process, where a correction ratio is applied (38). The notation Affine(*I*; *M*) signifies the affine operation that maps the floating image *I* to the fixed image using the affine matrix *M*. Moreover, *I* represents the output registrated image.

#### 2.2.2. Sampling weight map derived from brain tumor occurrence

**Figure 3:**
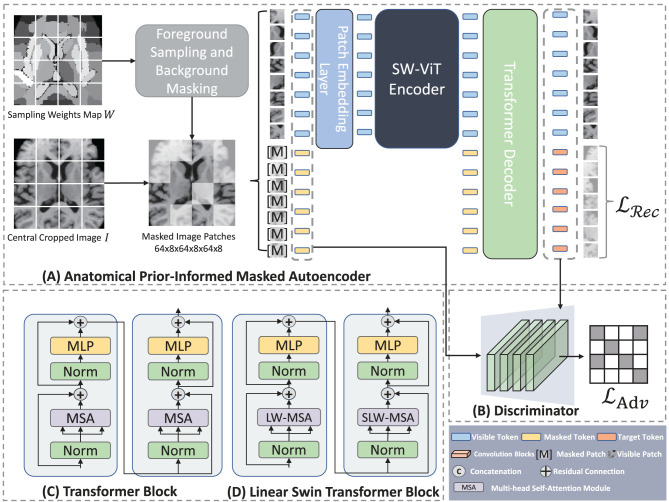
Architecture of Anatomical Prior-Informed Masked Autoencoder (API-MAE). **(A)** Is the architecture of Anatomical Prior-Informed Masked Autoencoder. **(B)** Is the Discriminator used for reconstruction. **(C)** If the Transformer block. **(D)** Is the linear Swin Transformer block.

We conduct a statistical analysis of enhanced tumor (ET) across BraTS21 dataset (39–41) and obatin a distribution map of ET occurrence in the SRI-24 standard space. To implement this analysis, we utilize a brain parcellation atlas building upon the parc116plus atlas (36). Some excessively small regions are merged into larger ones, resulting in 128 parcellation regions of the entire skull-stripped brain. To obtain the sampling probability of each voxel, the average sampling probability for each parcellation is defined as follows:


(2) $$P_{R_{i}}=\frac{\sum_{j}f_{i,j}}{V_{R_{i}}\cdot \sum_{i}\sum_{j}f_{i,j}}\;(i=1,2,\ldots ,128;j=1,2,\ldots ,N_{R_{i}})$$

where *R*_*i*_ represents the *i*-th brain parcellation, *P*_*R*_*i*__ denotes the average sampling probability per volume of region *R*_*i*_, *f*_*i, j*_ is the occurrence frequency of the ET region in the *j*-th voxel within the *i*-th parcellation, *V*_*R*_*i*__ represents the volume of *R*_*i*_, and *N*_*R*_*i*__ represents the number of voxel in *R*_*i*_. Consequently, the sampling weight map *W*, depicted in Figure 3, can be generated by assigning voxels within the parcellation region *R*_*i*_ the identical probability value *P*_*R*_*i*__.

### 2.3. Anatomical prior-informed masked auto-encoder

As shown in Figure 3, our proposed Anatomical Prior-Informed Masked AutoEncoder (API-MAE) consists of five components as follows: (1) Anatomical Prior-informed Masking, (2) Patch embedding, (3) Transformer Encoder, (4) Transformer Decoder, and (5) Discriminator.

#### 2.3.1. Anatomical prior-informed masking strategy

**Figure 4:**
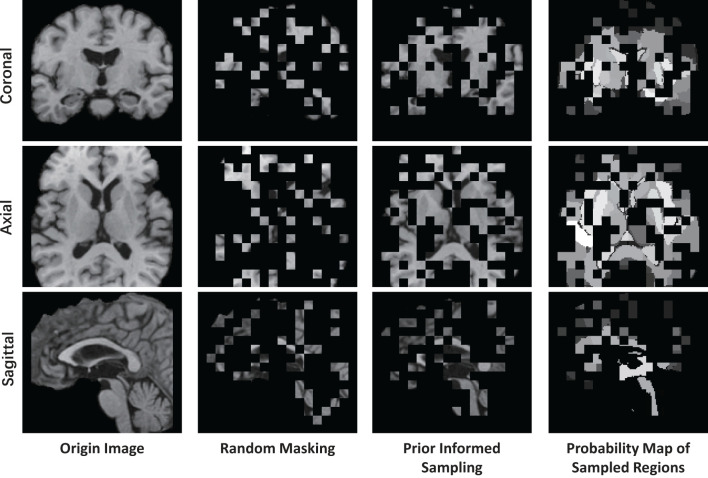
Example of visualizing a T1 MR image using different masking strategies with a masking ratio of 0.75. This image is center-cropped with a shape of 128 × 128 × 128, and each token has a patch size of 8 × 8 × 8.

Instead of the random masking strategy used in standard MAE pre-training, we propose a dedicated masking strategy to select informative patches based on the derived sampling weight map. The input image *I* and sampling weights map *W* are center-cropped with a size of 128, i.e., *I* ∈ ℝ^128 × 128 × 128^, *W* ∈ ℝ^128 × 128 × 128^. Subsequently, *I* and *W* are transformed into patches represented as $X={\left\{x_{i}\right\}}_{i=1}^{n}$ and $W={\left\{w_{i}\right\}}_{i=1}^{n}$, respectively. Here, *n* signifies the quantity of patches, and the patch size is configured at 8, a choice consistent with previous studies (35). This configuration leads to *n* = 16 × 16 × 16, aligning with the concept of vision transformers (12) splitting the 2D image into 16 × 16 tokens. The sample probability of each patch is determined by the probability vector $p={\left[p_{1},p_{2},\ldots ,p_{n}\right]}^{⊺}$, where $p_{i}=\sum_{j}w_{i,j}/\sum_{i,j}w_{i,j}$, and **w**_*i, j*_ denotes the sampling weight of the *j*-th voxel within the *i*-th patch corresponding to the voxels **x**_*i, j*_ of the image patch. Consequently, the visible patches that are fed into the encoder can be sampled as follows:


(3) $$\begin{array}{l} X_{\text{vis}}=\mathrm{Sampling}\left(X,p\right) \end{array}$$

where $X_{\text{vis}}={\left\{x_{i}\right\}}_{i=1}^{k}$ represents visible patches sampled from the original image patches X, and *k* = η·*n* represent the number of visible patches, η = 0.25 is the sampling ratio which aligned with the 75% masking ratio of MAE. The Sampling(X,p) operation involves utilizing a multinomial probability distribution with the probability vector ***p*** to select tokens from X for sampling, which then constitute the visible tokens. The sampling procedure is implemented using the multinomial API from PyTorch. As depicted in Figure 4, the prior-informed sampling maintains superior structural consistency compared to random masking, which is advantageous for the calculation of region-based sampling weights.

#### 2.3.2. Patch embedding

The input visible patches in $X_{\text{vis}}$ are first flattened into one-dimensional vectors, then mapped to the feature dimension *D* *via* learnable patch tokenizer g(·). The input of the transformer encoder **x**_enc_ is calculated as follows:


(4) $$x_{\text{enc}}=\text{g}(x_{i})+\text{PE}\in {\mathbb{R}}^{D}$$

where $x_{i}\in X_{vis}$, and PE is the sinusoidal positional encoding.


(5) $$\begin{array}{l} \text{PE}(pos,2i)=\sin\left(\frac{pos}{10000^{2i/D}}\right)\\ \text{PE}(pos,2i+1)=\cos\left(\frac{pos}{10000^{2i/D}}\right) \end{array}$$

where *pos* = 1, 2, …, *T* represents the token position, *i* represents the *i*-th dimension.

#### 2.3.3. Transformer encoder

We adopt a shifted window vision transformer, known as SW-ViT (35), as the transformer encoder in API-MAE. As shown in Figures 3C, D, the multi-head self-attention (MSA) in the original transformer block is replaced with linear window-based multi-head self-attention (LW-MSA) and shifted linear window-based multi-head self-attention (SLW-MSA) in the Swin transformer block. Both LW-MSA and SLW-MSA reduce parameters and computations among each head, which improves the network efficiency without significant accuracy loss. The transformer encoder serves as the feature extractor in API-MAE and the segmentation network. The output of the transformer encoder will undergo a linear projection to fit the higher feature dimension of the transformer decoder.

#### 2.3.4. Transformer decoder

We use a shallow transformer decoder to reconstruct the original image in API-MAE. The inputs to the decoder consist of both visible tokens and masked tokens with positional encodings. The output of the decoder is the reconstructed image tokens ${\hat{y}}_{i}$ for each input patch. The reconstruction loss function is the standard L2 loss:


(6) $${ℒ}_{\text{Rec}}=\frac{1}{2}\sum_{i}||{\hat{y}}_{i}-x_{i}{||}_{2},i=1,2,\ldots ,m$$

where **x**_*i*_ denotes the *i*-th image patch and *m* represents the number of masked tokens. It should be noted that only masked tokens are calculated for reconstructed loss.

#### 2.3.5. Reconstruction Discriminator

Recent advancements in self-supervised learning, such as DiRA (42), have demonstrated that the collaborative learning of self-supervised and adversarial tasks can lead to a more generalizable representation, encompassing fine-grained semantic representation. Moreover, discriminators have been proven beneficial for the masked autoencoder (32, 43). In API-MAE, we introduced a reconstruction discriminator, envisioning its potential synergistic effect when integrated into MAE decoder. This combination aims to enhance the learning representation and improve visual quality of the reconstructed output. The discriminator is constructed as a shallower convolutional neural network, comprising five convolutional layers tasked with distinguishing between the reconstructed and real images. The adversarial loss employed for the discriminator is represented as an L2 loss as follows:


(7) $${ℒ}_{\text{Adv}}=\frac{1}{2}\sum_{i}(||D(x_{i})-1{||}_{2}+||D({\hat{y}}_{i}){||}_{2}),\;i=1,2,\ldots ,n$$

where **x**_*i*_ is the *i*-th image patch, ${\hat{y}}_{i}$ is the corresponding reconstructed patch, and *n* is the token number of the original image. Thus, the total loss of API-MAE is a combination of reconstruction loss and adversarial loss as follows:


(8) $$\begin{array}{l} L_{\text{API-MAE}}=L_{\text{Rec}}+L_{\text{Adv}} \end{array}$$

### 2.4. Segmentation network

**Figure 5:**
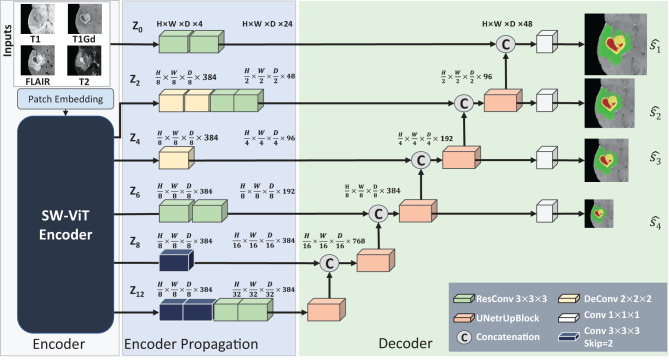
Architecture of the baseline segmentation network. This network is made up of three parts, i.e., the Encoder part for feature extraction, the Encoder Propagation part used for channel and spatial normalization and skip connection, and the Decoder parts used for upsampling and predicting the segmentation results. The convolution blocks with skip=2 in the Encoder Propagation part are used for downsampling, and the UNetrUpBlock used in the decoder part is used for upsampling and each block contains a deconvolution block and two residual convolution blocks.

After the pre-training of API-MAE, we discard the transformer decoder and keep the transformer encoder for the brain tumor segmentation task. The architecture of the segmentation network is shown in Figure 5. The segmentation network contains three parts as follows: (1) encoder, which contains patch embedding and transformer blocks, (2) encoder propagation, and (3) decoder. The patch embedding layer maps the input multi-parameter MRI (i.e., T1, T1Gd, T2-FLAIR, and T2 image) patches to the embedding features. The transformer blocks share the same architecture and are initialized with the pre-training weight of the transformer encoder in API-MAE. The encoder propagation and decoder parts utilize features from the original image (i.e., **z**_0_) and specific transformer layers (2nd, 4th, 6th, 8th, and last layer, i.e., **z**_2_, **z**_4_, **z**_6_, **z**_8_, **z**_12_) to propagate features and segment the image into three target classes as follows: whole tumor (WT), tumor core (TC), and enhanced tumor (ET). To obtain better segmentation, the segmentation network adopts cross-entropy and Dice loss with deep supervision as the segmentation loss as follows:


(9) $$\begin{array}{l} L_{\text{Seg}}=\overset{4}{\underset{i=1}{\sum }}\frac{1}{2^{i-1}}\cdot \left(\mathrm{CrossEntropy}\left(S_{i},{\hat{S}}_{i}\right)+\mathrm{Dice}\left(S_{i},{\hat{S}}_{i}\right)\right) \end{array}$$

where *i* represents the stage of deep supervision, ${\hat{S}}_{i}$ denotes the prediction of stage *i*, and *S*_*i*_ represents the ground truth resized to match the corresponding prediction.

## 3. Experiments

We pre-train the MAE model on an unannotated brain MRI dataset and evaluate the segmentation performance on an annotated brain tumor MRI dataset.

### 3.1. Datasets

#### 3.1.1. ADNI dataset

Alzheimer's Disease Neuroimaging Initiative (ADNI) dataset (44) is derived from a longitudinal multicenter study aimed at early detection and tracking of Alzheimer's disease (AD). In this study, we collected 7,945 skull-stripped T1 MR images and subsequently handpicked 6,415 images of superior visual quality for utilization in the pre-training dataset. This selection was made following a visual inspection of the registration results.

#### 3.1.2. BraTS21 dataset

The BraTS21 dataset (39–41) consists of 1,251 multi-parameter MRI scans. Each case includes four different modalities as follows: a) native (T1), b) post-contrast T1-weighted (T1Gd), c) T2-weighted (T2), and d) T2 Fluid Attenuated Inversion Recovery (T2-FLAIR) images, acquired from various protocols and scanners across multiple institutions. Each scan has been annotated by experienced radiologists with three different subregions as follows: enhancing tumor (ET), peritumoral edematous/invaded tissue (ED), and necrotic tumor core (NCR). In this study, we divide the 1,251 samples into training, validation, and testing sets at a ratio of 7:1:2, following previous studies (35).

### 3.2. Evaluation metrics

Both the volumetric metric dice similarity coefficient (DSC) and surface metric Hausdorff distance (HD) are used for performance evaluation. DSC quantifies the overlap between segmentation results and annotations in voxel space, while the 95_*th*_ percentile of Hausdorff distance (HD95) measures the distances between the segmentation surface and ground-truth surface. The calculation of HD95 is performed by the MedPy package using the analysis framework from nnFormer (45).

### 3.3. Implementation details

**Experimental settings**: All the experiments are implemented using the PyTorch 1.2 framework. We use 4 NVIDIA A100 GPUs (40 GB VRAM) for MAE pre-training and NVIDIA RTX3090 GPU (24 GB VRAM) for segmentation training and inference.

**Data preprocessing**: In the preprocessing section, we employ affine registration to align individual images with the standard space. Here, the cost function during the image registration optimization is correlation ratio (38). To prevent the registration results from being flipped upside down, we defined the rotation search space for affine registration as follows: [−30°, 30°] for X-axis rotation, [−30°, 30°] for Y-axis rotation, and [−180°, 180°] for Z-axis rotation. This configuration is aimed to emphasize rotation in the X-Y plane and prevent upside-down flipping along the Z-axis. It performed effectively with our dataset of 6,415 pre-training samples. The registration optimization and transformation processing were executed using the FLIRT (46) toolbox from FSL. Trilinear interpolation was utilized to compute the intensity of new voxels during affine mapping. For the pre-training data, we employ the MONAI (47) library for data normalization and cropping. Additionally, we utilize the segmentation data preprocessing pipeline provided by nnUNet (7), to handle the multi-modality segmentation data.

**Model architecture**: In API-MAE, the transformer encoder contains 12 layers of linear swin transformer blocks with a feature dimension *D* = 384. The transformer decoder comprises 8 layers of vanilla transformer blocks with a feature dimension of 384. The discriminator consists of four convolution blocks with a kernel size of *k* = 3 and a convolution block with a kernel size of *k* = 1. In the segmentation network, the weights of encoder propagation and decoder parts are initialized with the He initialization (48).

**Model training**: For MAE training, the AdamW optimizer with a batch size of 12 is trained for 300 epochs. The initial learning rate is 1e-3. Weight decay of 5e-2 is also adopted for model regularization. For the segmentation procedure, we apply the (45) training framework and default parameter for 1,000 epochs.

## 4. Results

### 4.1. Pre-training results of anatomical prior-informed MAE

**Figure 6:**
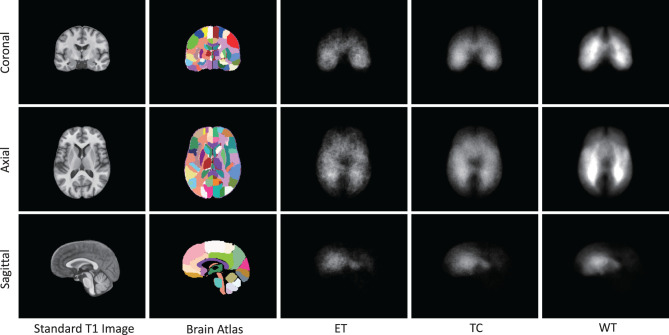
The occurrence frequency in SRI-24 standard space among 1,251 cases from BraTS21 dataset. The five columns represent the standard brain T1 MR image, brain atlas (enhanced parc116 plus) in SRI-24 Space, Enhanced Tumor (ET) occurrence, Tumor Core (TC) Tumor occurrence, and Whole Tumor (WT) occurrence, respectively.

As presented in Figure 6, we note distinct differences in the spatial distribution of tumor occurrence within the SRI24 space. Specifically, gliomas are more frequently observed in the white matter regions of the middle and posterior sections of the brain, with comparatively lower frequencies in the brainstem and cerebellar regions. Table 1 shows the normalized probability of tumor occurrence among all 128 brain parcellations. Considering that ET is the most challenging region to segment, we employ the probability of the ET region for probabilistic masking.

**Table 1:** The normalized occurrence of tumor regions within different brain parcellations in enhanced SRI-24 atlas analyzed from 1,251 training cases of the BraTS21 dataset.

**Atlas No**.	**Occurrence (**‰**)**			**Atlas No**.	**Occurrence (**‰**)**			**Atlas No**.	**Occurrence (**‰**)**			**Atlas No**.	**Occurrence (**‰**)**		
	**ET**	**TC**	**WT**		**ET**	**TC**	**WT**		**ET**	**TC**	**WT**		**ET**	**TC**	**WT**
1	4.752	6.232	7.804	33	9.670	9.221	8.695	65	6.924	6.237	6.478	97	0.234	0.371	0.451
2	6.076	7.487	9.324	34	10.018	8.920	9.074	66	5.039	5.836	6.568	98	0.566	0.627	0.660
3	4.488	5.667	6.900	35	8.562	6.641	5.224	67	5.838	5.624	5.486	99	0.432	0.359	0.365
4	7.042	7.193	7.949	36	7.492	5.694	4.343	68	5.379	4.854	4.737	100	0.346	0.406	0.432
5	4.258	4.047	4.198	37	22.961	19.311	16.840	69	3.144	3.872	4.267	101	0.511	0.250	0.221
6	2.253	3.679	3.666	38	20.812	18.402	15.837	70	3.148	3.553	4.698	102	0.006	0.020	0.108
7	5.058	5.902	7.232	39	10.437	8.829	8.304	71	17.277	18.222	16.062	103	0.443	0.244	0.238
8	7.983	8.575	8.887	40	8.959	8.447	7.602	72	17.632	19.147	18.173	104	0.077	0.102	0.154
9	4.697	4.000	4.069	41	20.511	17.402	15.713	73	21.153	20.498	20.578	105	0.234	0.234	0.324
10	4.302	4.591	4.235	42	19.540	18.639	16.355	74	22.365	23.167	22.596	106	0.297	0.236	0.338
11	10.660	11.250	12.304	43	3.381	3.224	3.248	75	16.388	16.856	17.372	107	0.000	0.048	0.104
12	12.720	13.980	14.909	44	2.699	2.668	2.584	76	19.246	19.615	18.951	108	0.109	0.091	0.130
13	6.800	6.684	7.671	45	5.139	4.690	4.523	77	10.394	10.765	11.793	109	0.571	0.502	0.674
14	8.131	8.618	8.160	46	4.073	3.794	3.657	78	16.606	14.655	15.036	110	0.332	0.496	0.603
15	8.506	7.635	7.178	47	1.932	1.842	1.673	79	20.442	19.811	21.291	111	0.649	0.531	0.665
16	6.499	6.783	6.170	48	2.241	1.949	1.706	80	23.945	25.019	22.553	112	0.385	0.244	0.307
17	13.663	14.048	16.044	49	6.592	5.891	5.982	81	13.914	14.137	15.073	113	0.513	0.320	0.336
18	17.307	17.339	17.131	50	4.727	4.686	5.243	82	15.253	15.150	14.987	114	0.212	0.342	0.395
19	3.260	4.923	5.407	51	4.827	4.206	4.233	83	18.236	16.137	15.620	115	17.195	15.945	14.050
20	3.897	4.862	5.722	52	4.906	4.636	4.816	84	13.306	13.089	12.785	116	8.231	8.323	9.206
21	7.835	8.926	9.186	53	1.949	1.836	1.902	85	11.423	10.749	11.322	117	6.310	7.011	9.164
22	7.424	9.238	8.825	54	2.332	2.017	1.918	86	9.425	10.035	10.850	118	6.761	7.458	9.569
23	5.723	7.193	6.791	55	7.666	6.745	6.341	87	11.912	10.302	11.184	119	19.636	18.910	16.956
24	8.352	8.358	8.053	56	6.645	6.265	5.671	88	9.728	8.737	8.556	120	21.037	20.192	18.556
25	5.593	5.984	6.282	57	5.545	6.162	7.175	89	9.253	8.937	9.267	121	2.881	7.415	6.751
26	5.275	5.768	6.391	58	7.017	7.076	8.576	90	7.208	7.937	7.986	122	3.018	7.112	6.942
27	3.380	3.943	3.972	59	6.488	6.475	6.568	91	0.382	0.253	0.251	123	15.209	15.058	16.662
28	2.602	3.767	3.784	60	5.566	5.746	7.283	92	0.046	0.066	0.137	124	16.987	16.541	18.001
29	22.308	22.074	20.767	61	7.063	6.733	6.843	93	0.425	0.228	0.177	125	7.655	7.821	7.360
30	22.077	23.383	20.966	62	7.861	7.588	9.150	94	0.000	0.033	0.110	126	7.681	8.149	7.542
31	15.676	14.889	13.405	63	6.903	6.783	7.793	95	0.233	0.461	0.587	127	3.154	3.684	3.294
32	17.266	15.530	13.671	64	8.235	7.420	7.827	96	0.462	0.396	0.585	128	3.571	4.070	3.562

The occurrence is expressed in permillage format. ET, enhanced tumor; TC, means tumor core; WT, the whole tumor.

**Figure 7:**
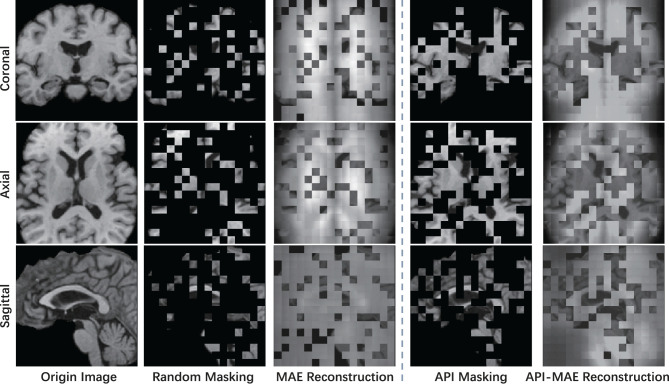
The visible example of masking images and the reconstruction results of MAE and API-MAE. The five columns represent the origin brain T1 MR image, the random masking strategy used in MAE, the masking image generated from API token sampling, and the reconstruction results of API-MAE, respectively.

The masking and reconstruction results are shown in Figure 7. It can be observed that random masking tends to distribute masked patches uniformly across the entire image, whereas our proposed weighted sampling strategy enables concentration on more valuable, concentrated, and relatively contiguous regions. The disruption of contextual information in random masking makes the reconstruction task challenging and results in a blurry reconstructed image. In contrast, the proposed weighted sampling method can maintain the integrity of semantic regions, allowing for better reconstruction results.

### 4.2. Segmentation results on BraTS21 dataset

#### 4.2.1. Segmentation performance on BraTS21 dataset

To validate the effectiveness of the proposed SSL pre-training approach in downstream segmentation task, we conducted validation experiments using the BraTS21 dataset. The downstream brain tumor segmentation network is initialized with the pre-trained API-MAE encoder weights and subsequently fine-tuned using the BraTS21 dataset. We conducted a comparison of our method against several transformer-based models, including nnFormer (45), TransBTS (16), and UNETR (13) without pre-training. Additionally, we compared against several SSL pre-training methods used in medical imaging, namely, 3D-RPL and 3D-Jig (49), as well as the current state-of-the-art ASA in brain tumor segmentation (35).

As shown in Table 2, we observed that the pre-trained models demonstrate better performance, and our proposed API-MAE achieved the best performance in terms of the Dice similarity coefficient (DSC) metrics for whole tumor (WT) and tumor core (TC) and the best average performance of all three regions.

**Table 2:** Efficiency analysis.

**Metric**	**nnFormer**	**TransBTS**	**UNETR**	**SW-ViT**
FLOPs (G)	271.64	527.46	2141.32	860.03
Params (M)	37.48	30.62	91.04	85.29
CPU inference time (s)	1.425	16.011	21.953	5.030
GPU inference time (s)	0.010	0.006	0.008	0.106

FLOPs stands for Floating Point Operations, which are recorded in units of gigaflops. Params refers to the learnable parameters of different network architectures, recorded in units of millions. Inference time is computed using an input tensor with dimensions of 2 × 128 × 128 × 128.

#### 4.2.2. Ablation study on masking strategies

To evaluate the effectiveness of our proposed masking strategy, we conduct an ablation study on different MAE masking strategies. The comparison methods include the baseline without pre-training, MAE pre-trained with random masking, and our proposed API-MAE pre-trained with anatomical prior-informed masking strategy. Table 3 shows that our proposed API-MAE showed improved performance for all regions compared with vanilla MAE and baseline. This demonstrates the effectiveness of our anatomical prior-informed masking compared with the random masking strategy. However, the marginal improvement indicates that in the presence of enough annotated data (more than 1,000 cases in BraTS21), transformer-based models already achieve satisfactory performance, and the benefit of pre-training is not substantial.

**Table 3:** Ablation study on the segmentation performance trained on the BraTS21 dataset.

**Methods**	**DSC (%)**↑				**HD95 (mm)**↓			
	**WT**	**TC**	**ET**	**Mean**	**WT**	**TC**	**ET**	**Mean**
Baseline	93.96	91.06	85.83	90.28	**3.700**	3.600	**2.566**	3.288
MAE	93.84	90.78	86.20	90.27	3.977	3.619	2.758	3.451
Ours	**94.07**	**91.47**	**86.53**	**90.69**	3.825	**3.172**	2.680	**3.225**

DSC means the Dice similarity coefficient, and HD95 means the 95th percentile Hausdoff distance. ↑ indicates higher is better and ↓ indicates lower is better. Bold indicates the best performance.

#### 4.2.3. Data-efficiency analysis

To validate the data efficiency of our pre-trained model, we further train the segmentation model on a small subset of the whole training dataset. We randomly sampled 100 cases from the original training cases, while the validation and testing sets were kept the same as the whole dataset. The compared methods include the baseline without pre-training, MAE pre-trained with random masking, and our proposed API-MAE pre-trained with anatomical prior-informed masking strategy. The sampling process is repeated four times to mitigate the selective bias.

**Figure 8:**
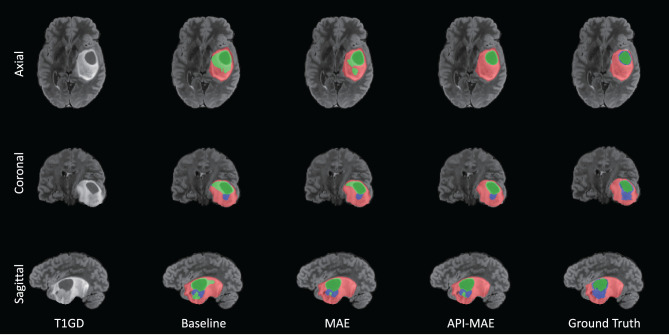
Example of tumor segmentation results from a testing image with 100 training cases. The three rows are from the axial, coronal, and sagittal views. The green region represents the necrotic tumor core (NCR), the blue region represents the Gd-enhancing tumor (ET), and the red region represents the peritumoral edematous/invaded tissue (ED).

The segmentation results on the small training set are shown in Table 4. It is observed that MAE pre-training benefits the segmentation performance and improves the model robustness in most scenarios. The improvement by pre-training is more prominent in this small-dataset setting compared with the whole dataset. The best segmentation performance for ET and TC regions is obtained by API-MAE, in terms of DSC metrics, which matches the purpose of using ET occurrence map for weighted sampling. As shown in Figure 8, training with the MAE paradigm tends to reduce the erroneous falsely predicted regions and reduce the prediction error of ET regions, particularly in difficult-to-segment regions.

**Table 4:** Comparison of model performance trained with 100 cases sampling from BraTS21 dataset.

**Metric**	**Methods**	**WT**	**TC**	**ET**	**Mean**
2]*DSC (%)↑	Baseline	91.83 ± 0.16	87.43 ± 1.88	83.11 ± 1.44	87.46
	MAE	**91.96** **±** **0.03**	87.89 ± 0.53	83.75 ± 0.59	87.87
	API-MAE	91.95 ± 0.19	**88.02** **±** **0.68**	**84.25** **±** **0.67**	**88.07**
2]*HD95 (mm)↓	Baseline	6.489 ± 0.426	5.563 ± 0.377	3.985 ± 0.226	5.346
	MAE	**6.262** **±** **0.358**	5.513 ± 0.722	4.093 ± 0.692	5.289
	API-MAE	6.285 ± 0.694	**4.979** **±** **0.424**	**3.856** **±** **0.384**	**5.040**

Results from four independent sampling processes are reported with mean±std.↑ indicates higher is better and ↓ indicates lower is better. Bold indicates the best performance.

To further investigate the efficiency of proposed method, we conducted an efficiency analysis of the segmentation phase for the methods, as shown in Table 5. Since different SSL methods share the same segmentation network, specifically SW-ViT, the variations in performance arise from the encoder weights inherited from diverse SSL pre-training tasks. This comparison involves distinct network architectures, namely, nnFormer, TransBTS, UNETR, and SW-ViT. All the methods were reproduced using the original code on a local server equipped with an AMD Ryzen 9 5900X CPU (3.7 GHz), 128 GB RAM (DDR4 2400MT/s), and an NVIDIA RTX3090 GPU. For fair comparison, we modified UNETR by adjusting its input channels to 4 and configuring the patch size as 8 × 8 × 8, in alignment with SW-ViT. The computation consumption was calculated utilizing the thop package. This process entails inputting a tensor with dimensions of 2 × 128 × 128 × 128 into the network for computation and the standard segmentation procedure.

**Table 5:** Comparision results on BraTS21 dataset.

**Methods**	**DSC (%)**↑				**HD95 (mm)**↓			
	**WT**	**TC**	**ET**	**Mean**	**WT**	**TC**	**ET**	**Mean**
nnFormer (45)	91.46	87.42	82.22	87.03	10.15	9.59	16.78	12.17
TransBTS (16)	92.06	88.20	79.46	86.57	4.98	4.86	16.32	8.72
UNETR (13)	92.12	88.32	79.61	86.68	4.91	4.67	16.32	8.63
3D-RPL (49)	93.92	90.13	85.92	89.99	3.74	3.98	13.71	7.14
3D-Jig (49)	93.87	90.14	86.01	90.01	3.85	3.94	11.79	6.53
ASA (35)	94.03	90.29	**86.76**	90.36	**3.61**	3.78	10.25	5.88
Ours	**94.07**	**91.47**	86.53	**90.69**	3.82	**3.17**	**2.68**	**3.23**

DSC means the Dice similarity coefficient, and HD95 means the 95th percentile Hausdoff distance. ↑ indicates higher is better and ↓ indicates lower is better. Bold indicates the best performance and the results of previous studies are adopted from (35).

Combining the data from Tables 2, 4, we observe that nnFormer exhibits the best inference efficiency. This superiority can be attributed to the dimension of the embedding feature in the Transformer module of the network, which is [96, 192, 384, 768]. In contrast, other Transformer models often have embedding feature dimensions of 384 or 768. This relatively shallower transformer architecture contributes to its enhanced computational efficiency. However, it may result in slightly lower segmentation performance. Higher segmentation accuracy can be achieved in both WT and TC components in models with increased transformer layers. However, when using a high-layer transformer encoder such as UNETR, the number of floating point operations (FLOPs) and learnable parameters will increase rapidly. While the SW-ViT could reduce the FLOPs and parameters with the help of shifted window-based linear transformer modules. Enhanced with SSL pre-training tasks, particularly our proposed API-MAE, the methods using SW-ViT obtain the best segmentation performance while maintaining a favorable balance in terms of segmentation time consumption. Due to the presence of certain operations within the network architecture that do not parallelize efficiently during GPU computation, the proposed method does not achieve optimal computational efficiency on the GPU. However, the proposed method could attain decent CPU time consumption, which maintains a reasonable balance between accuracy and efficiency.

## 5. Discussion

Recently, transformer-based models have emerged as state-of-the-art methods for 3D medical image segmentation, owing to their superiority in modeling long-range dependencies and leveraging global contextual information over fully convolutional neural networks. However, such methods often rely on a vast of training data for network optimization. A major challenge in training such models is the limited availability of annotated data. In this study, we address this challenge by utilizing 6,415 unannotated T1-weighted MR images from the ADNI dataset for pre-training. Our approach consistently improved the segmentation accuracy in scenarios with both large and small training sets. Although only T1-weighted images are used for pre-training, the learned weights benefit the downstream brain tumor segmentation on multi-parameter MRI. This highlights the potential of pre-training for improved medical image segmentation.

The MAE used in computer vision typically employs random masking with a high masking ratio of 0.75 and utilizes 25% unmasked patches for encoder training. The high masking ratio can lead to the loss of contextual information in high-dimensional medical images, making image reconstruction challenging and potentially affecting the learning of generalizable features. Therefore, it is important to consider tailored sampling strategies that take into account the specific characteristics and requirements of the task at hand. In this study, we introduce an anatomical prior-informed masking strategy, where brain regions with higher tumor occurrence are more frequently sampled for pre-training. The experiments demonstrate that our proposed pre-training method enhances the performance of brain tumor segmentation, which outperforms other self-learning approaches. This indicates that incorporating anatomical priors into the pre-training stage leads to performance improvements in downstream tasks.

Additionally, our anatomical prior-informed sampling strategy can be considered as an attention mechanism in selecting valuable and task-related patches for MAE pre-training. In general, attention mechanisms usually help models filter out high-value information from large amount of data, thereby improving computational efficiency and performance and making computing more precise and efficient. Given a large number of image patches in the unannotated dataset, it is important to let the pre-training process attend the informative patches. By incorporating the tumor occurrence rate and brain template into the construction of an attentive sampling strategy, our approach integrates anatomical priors with masked image modeling pre-training. This enables efficient sampling and the most use of unannotated data.

There are some limitations of this study. Our proposed method requires the pre-registration of the sampling weighting map for each individual, a process typically executed on the CPU and incurring a time cost. In future study, this procedure can be expedited through the utilization of deep learning-based networks, enabling accurate and rapid registration. We showcase the advantage of integrating anatomical priors during the pre-training stage, leveraging only tumor occurrence information. In future, the exploration of more advanced anatomical priors, such as symmetric brain structure or active learning strategies (50), holds potential for further investigation.

## 6. Conclusion

In this study, we introduce a novel pre-training technique for brain tumor segmentation utilizing transformer networks. This technique involves the integration of an anatomical prior-informed masking strategy into the masked image modeling process. Informative image patches from brain parcellations with higher tumor occurrence are sampled more frequently, facilitating the mask autoencoder to focus on the regions of interest. The proposed approach demonstrates promising performance in the brain tumor segmentation task, surpassing compared self-learning methods.

## Data availability statement

The raw data supporting the conclusions of this article will be made available by the authors, without undue reservation.

## Author contributions

KW, HW, MW, and SW: main idea and study design. ZL and MP: organized the database, data inspection, and analysis. KW, ZL, and SW wrote the first draft of the manuscript. SL: built the initial code of the network training framework. MW, SW, and ZS: supervised, supported, and revised the manuscripts. All authors contributed to the manuscript revision, read, and approved the submitted version.
